# Protocol for a systematic literature review of smartphone apps to support the self-management of rheumatic and musculoskeletal diseases: development strategies, theoretical underpinnings and barriers to engagement

**DOI:** 10.1186/s13643-023-02276-4

**Published:** 2023-07-29

**Authors:** Rosemarie Barnett, Christopher Clarke, Raj Sengupta, Peter C. Rouse

**Affiliations:** 1 Department for Health, University of Bath, Bath, UK; 2 Royal National Hospital for Rheumatic Diseases, Royal United Hospitals Bath NHS Foundation Trust, Bath, UK; 3 Department of Computer Science, University of Bath, Bath, UK

**Keywords:** Rheumatic and musculoskeletal diseases, Smartphone apps, Digital health technologies, Self-management

## Abstract

**Background:**

Rheumatic and musculoskeletal diseases (RMDs) cause significant burden to the individual and society, requiring lifelong management and specialist healthcare resource use. Costing over 200 billion euros per year in Europe, RMDs are the most expensive of all diseases for European healthcare systems. The incidence and burden of RMDs are projected to rise with the ageing global population and increase in sedentary, obesogenic lifestyles. In parallel, there is a global crisis in the rheumatology workforce, whereby capacity to deliver specialist care is being exceeded by demand. Pervasive, scalable mobile health technologies, such as apps, are being developed to support the self-management of RMDs and reduce pressure on healthcare services. However, it is unknown whether these apps are informed by theory or their use supported by an appropriate evidence base. The purpose of this review is therefore to provide a comprehensive overview of the development strategies, interventional components and theoretical underpinnings of existing smartphone apps, designed to support the self-management of RMDs.

**Methods:**

Searches will be conducted within PubMed, Scopus, Web of Science, Embase, MEDLINE and PsycINFO. Reference lists and citing articles of the included studies will be searched. Identified publications will be screened for eligibility by two independent reviewers. Any discrepancies between reviewers will be resolved by consensus, with input from a third reviewer if required. Data will be extracted on study designs, methods, populations, setting, utilised theoretical frameworks, intervention components, behaviour change techniques, methods to evaluate effectiveness and barriers/facilitators to intervention engagement. Exploratory outcomes include reported effectiveness, acceptability and usability. A systematic, narrative synthesis of evidence will be presented. If appropriate (depending on quality and pool of evidence identified), qualitative meta-summary techniques will be used to combine and summarise qualitative findings regarding barriers/facilitators to intervention engagement.

**Discussion:**

The results of this systematic literature review will provide insights for healthcare professionals, researchers, app designers and policy makers, to inform future development and implementation of smartphone apps to support self-management of RMDs. Evidence gaps for future research will be identified. Findings will be disseminated through a final manuscript/publication of results and via a conference abstract, patient organisations and social media.

**Systematic review registration:**

PROSPERO CRD42022359704.

**Supplementary Information:**

The online version contains supplementary material available at 10.1186/s13643-023-02276-4.

## Background and rationale

Rheumatic and musculoskeletal diseases (RMDs) comprise a highly heterogeneous and prevalent group of noncommunicable diseases, primarily impacting the musculoskeletal system [1–3]. The Global Burden of Disease Study estimated that in 2017, there were approximately 1.3 billion people living with musculoskeletal disorders worldwide; 121.3 thousand deaths and 138.7 million disability-adjusted life years attributed to these conditions [4]. There are over 200 inflammatory or degenerative RMDs [2]. Some are prevalent and well-known such as rheumatoid arthritis (RA), osteoarthritis (OA) and gout. Others such as systemic lupus erythematosus are less common but still cause significant morbidity and mortality [2]. In the USA, the overall lifetime risk for developing an inflammatory RMD such as RA, gout, lupus or spondyloarthritis (SpA) has been estimated as 1 in 12 for women and 1 in 20 for men [5]. Conservative estimates from the United Nations suggest that approximately 15% of the global population live with OA or degenerative joint disease (mechanical/degenerative RMDs) [6]. These estimates suggest that by 2050, over 130 million people worldwide will live with OA, and approximately 40 million will be severely disabled by the condition.

RMDs are a leading cause of disability globally, with a profound impact on quality of life due to chronic pain, reduced function, social exclusion and loss of employment or reduced productivity [3, 7–10]. Although RMDs are frequently associated with arthritis (joint inflammation), commonly considered a disease of ageing, many RMDs, including disabling forms of arthritis, can occur in children [2]. RMDs can affect all ages and genders (prevalence varying per specific condition) and can involve other tissues, the skin and internal organs [11]. They are often chronic conditions, leading to significant burden both to the individual and society, requiring lifelong management and specialist healthcare resource use [2, 12]. The economic burden of RMDs is substantial, costing over 200 billion euros per year in Europe [8, 11]. This is expected to rise with increased diagnosis of RMDs due to the ever-growing and ageing global population, compounded by increasing sedentary lifestyles and a rise in obesity [8, 12]. By 2040, the number of adults diagnosed with arthritis in the USA is projected to increase by 49% to 78.4 million [13].

In parallel, we are seeing a global crisis in the rheumatology workforce [14]. Capacity to deliver specialist care is being exceeded by demand, further exacerbated by the COVID-19 pandemic [15]. Reports in Canada, the USA and UK highlight a significant shortage of rheumatologists [15–18]. In the USA, the 2015 American College of Rheumatology (ACR) workforce study projected that by 2030, demand for rheumatology services will exceed available resources by 102%, in part due to a 25% decline in adult rheumatology providers (physicians, specialist nurses and physician assistants) and anticipated retirement of nearly 50% of the current workforce [17]. The recent British Society for Rheumatology (BSR) report paints a particularly grim picture [15, 19]. The 2021 publication reports a dangerous lack of consultants, specialist nurses and poor access to allied health professionals, resulting in unacceptable, unnecessary consequences for people living with RMDs in the UK, such as progressively worse health and debilitating pain [15]. Responses from 80% of UK departments to BSR data requests conducted as part of this report indicated that vacancy rates for rheumatology posts exceeded 49% in some areas and were greatest among consultants [15, 19]. Post-pandemic workload pressures have led to further challenges, particularly regarding provision of educational and non-pharmacological treatment, making good long-term self-management more difficult for patients [20]. Healthcare systems must adapt, and multifaceted approaches will be critical to resolve these challenges [14].

Telemedicine has frequently been cited as a strategy to overcome workforce challenges and improve quality of care for people living with RMDs [14, 21–23]. Telehealth interventions can make use of digital technologies across all stages of the patient journey and have been suggested to improve healthcare access and outcomes, reduce demands on overstretched facilities and make the health sector more resilient, particularly in the context of chronic disease [24, 25]. The European Alliance of Associations for Rheumatology (EULAR, formerly European League Against Rheumatism) have recently recommended that telehealth should be considered for non-pharmacological interventions, including those delivering education and self-management strategies [21]. In particular, EULAR have noted the ability of pervasive, scalable mobile health technologies such as apps, to support self-management and encourage/allow patients to take a more active role in their health, with the number of smartphone users worldwide surpassing 6 billion in 2022 [26, 27]. However, the potential of smartphone apps to support self-management in rheumatology is yet to be fully realised [28–33]. In part, we hypothesise this may be due to a lack of application of motivational theory during app development and, thus, a lack of understanding regarding the causal pathways between the designed intervention, underpinning mechanisms of motivational behaviour change and intended outcomes. Indeed, adoption of self-management behaviours or digital interventions is dependent on an individual’s motivation to engage in a change process. Human motivational theory should therefore be leveraged to better understand and optimise intervention development and deployment [34–36].

The last decade has borne witness to tremendous innovation in rheumatology, including an explosion in available healthcare apps to support people with RMDs in their self-management [28–31, 37–47]. However, it is not yet known whether these apps are informed by theory or supported by an appropriate evidence base. A recent (pre-pandemic) systematic literature review by EULAR (published end of 2019, search conducted up to December 2017 [47]) identified moderate to low quality apps, with limited involvement of healthcare professionals in app development. A review is yet to be conducted that includes a comprehensive evaluation of how motivational theories and published/validated development frameworks are being utilised (if at all) to inform app development, intervention components and content. Furthermore, this is a rapidly developing field — whereby regular updates regarding existing apps and development processes are needed, particularly given the recent boom in digital health innovation, catalysed by the COVID-19 pandemic [48–50]. There is therefore a need for an up-to-date literature review of current available apps, in addition to a detailed exploration of their theoretical underpinnings. As per the PICO (population, intervention, comparator, outcome) framework, this systematic literature review will aim to explore how smartphone apps (*intervention*) are being developed and utilised in rheumatology, to support people with RMDs (*population*) in their self-management. A comprehensive overview of currently utilised theoretical frameworks, intervention components, behaviour change techniques and barriers/facilitators to intervention engagement (*outcomes*) will be presented. We will also explore how effectiveness has been assessed. A comparator (as per the PICO framework) is therefore not applicable. This protocol documents the proposed methodology of the systematic literature review, to ensure methodological rigour and transparency, reduce bias and avoid duplication of research efforts.

## Objectives

The primary research question is as follows: How are smartphone applications (smartphone “apps”) being developed and utilised in rheumatology to support self-management? Specifically, what are the:

- Theoretical frameworks (e.g. self-determination theory, self-efficacy theory, activity theory, persuasive systems design) being utilised to promote (a) self-management and (b) app engagement for people living with RMDs?
- Intervention components (targeted behaviours, behaviour change techniques — BCTs, app features) and proposed mechanisms of action (e.g. theoretical constructs the BCTs are believed to modify)?
- Development strategies and processes (e.g. person-based approach, behaviour change wheel, iterative co-design with users, alignment with EULAR points to consider when developing and evaluating smartphone apps for self-management [31])?
- Methods (and outcome measures) utilised to evaluate effectiveness?
- Key facilitators and barriers to intervention engagement for people living with RMDs?

The exploratory research question (dependent upon findings from primary research question, point d above) is as follows: How acceptable, usable and effective are smartphone applications (smartphone “apps”) for supporting self-management in people living with RMDs?

## Methods

This protocol has been developed in alignment with the 2015 PRISMA-P Preferred Reporting Items for Systematic review and Meta-Analysis Protocols: recommended items to address in a systematic review protocol [51, 52]. Guidance from Petticrew et al. regarding synthesising evidence on complex interventions has been followed [53].

This protocol is registered on the PROSPERO database with registration number CRD42022359704. To establish the quality of our protocol, we have completed the PRISMA-P 2015 checklist, Additional file 1. If required, amendments to the protocol will be documented (including date, what was changed, rationale) and added to the PROSPERO registration record. Amendments will also be reported in the final systematic review results publication/report.

### Eligibility criteria

#### Types of studies

All original research articles will be included (no publication date restriction), for example randomised controlled trials, nonrandomized intervention studies, observational studies, case series, case reports and feasibility studies or protocols. Review articles, letters to editors, editorials, commentaries and guidelines will be excluded. Abstracts and conference proceedings will be excluded if no full-text journal article is found, as these articles would not provide sufficient information for assessment. Every effort will be made to find English-language versions of an article. However, if an English-language version of the article cannot be located, the article will be excluded due to limited resources available for translation purposes (see section regarding “Meta-biases”).

#### Types of participants

We will include studies where participants are individuals (humans) diagnosed with a chronic RMD. Participants with acute injury, or with an intervention prescribed for acute self-management pre-/post-surgery, will not be included. Non-specific diagnoses such as low back pain will also not be included.

#### Types of intervention

Interventions must be designed to promote long-term self-management via a native smartphone application (for android/Apple) or progressive web application. The intervention must be designed for primarily remote use (or blended), rather than in an inpatient/outpatient setting.

### Electronic searches

Searches will be conducted within PubMed, Scopus, Web of Science, Embase, MEDLINE and PsycInfo. The search strategy was first developed within PubMed, utilising search terms outlined in the 2019 EULAR paper by Najm et al. and with guidance from an experienced librarian [47]. Three key concepts and initial search terms were defined as follows: (1) smartphone app, (2) self-management and (3) RMDs (see Table 1). These concepts were then updated and refined, to include additional headings/ searches, as appropriate for each database to ensure that no relevant publications are missed. No restrictions will be given regarding publication date. Searches will be re-run prior to the final analyses, and any additional eligible studies published more recently than the date of the initial search will be identified and retrieved for inclusion. Methods such as screening the reference lists of included articles, and the follow-up of citing articles, will further minimise the chance of overlooking valuable publications.

**Table 1:** PubMed key concepts and initial search terms, including MeSH headings

**Key concept 1: Smartphone app**
"mobile app*" OR "smartphone*" OR "mobile technolog*" OR "mobile health*" OR "mhealth*" OR “m-health*” OR "Smartphone"[Mesh] OR "Mobile Applications"[Mesh] OR "Cell Phone"[Mesh] OR "telemedicine"[MeSH Terms] OR "telemedicine" OR "smartphoneassisted" OR "smartphonebook" OR "smartphones4water" OR "pedometer*" OR "accelerometer*"
**Key concept 2: Self-management**
"self-care" OR "self-monitoring" OR "Self-Management"[Mesh] OR "Disease Management"[Mesh] OR "Pain Management"[Mesh] OR "Medication Therapy Management"[Mesh] OR "therapy" [Subheading] OR "Blood Pressure Monitoring, Ambulatory"[Mesh] OR "manage*" OR "monitor*" OR ("organization and administration"[MeSH Terms] OR ("organization"[All Fields] AND "administration"[All Fields]) OR ("organisation"[All Fields] AND "administration"[All Fields]) OR ("disease"[All Fields] AND "management"[All Fields]) OR care[All Fields] OR (patient reported outcome[All Fields] OR patient reported outcomes[All Fields]) OR (patient reported outcome measure[All Fields] OR patient reported outcome measurement[All Fields] OR patient reported outcome measurements[All Fields] OR patient reported outcome measures[All Fields]) OR ("pain"[MeSH Terms] OR "pain"[All Fields]) OR ("fatigue"[MeSH Terms] OR "fatigue"[All Fields]) OR ("Infections"[Mesh]OR "infection"[All Fields] OR "infections"[All Fields]) OR (morning[All Fields] AND stiffness[All Fields]) OR ("affect"[MeSH Terms] OR "affect"[All Fields] OR "mood"[All Fields]) OR ("depressive disorder"[MeSH Terms] OR ("depressive"[All Fields] AND "disorder"[All Fields]) OR "depression"[All Fields] OR "depression"[MeSH Terms]) OR workout[All Fields] OR "disease activity"[All Fields] OR (("disease"[MeSH Terms] OR "disease"[All Fields]) AND ("motor activity"[MeSH Terms] OR ("motor"[All Fields] AND "activity"[All Fields]) OR "activity"[All Fields])) OR ("pharmaceutical preparations"[MeSH Terms] OR ("pharmaceutical"[All Fields] AND "preparations"[All Fields]) OR "medication"[All Fields]) OR ("motor activity"[MeSH Terms] OR “activity"[All Fields]))
**Key concept 3: RMDs**
"musculoskeletal diseases"[MeSH Terms] OR "Arthritis"[Mesh] OR ("musculoskeletal"[All Fields] AND "diseases"[All Fields]) OR ("osteoarthritis"[MeSH Terms] OR "osteoarthritis"[All Fields]) OR ("connective tissue diseases"[MeSH Terms] OR ("connective"[All Fields] AND "tissue"[All Fields] AND "diseases"[All Fields])) OR ("rheumatic diseases"[MeSH Terms] OR ("rheumatic"[All Fields] AND "diseases"[All Fields])) OR ("lupus vulgaris"[MeSH Terms] OR ("lupus"[All Fields] AND "vulgaris"[All Fields]) OR "lupus"[All Fields]) OR sjorgen[All Fields] OR ("scleroderma, systemic"[MeSH Terms] OR ("scleroderma"[All Fields] AND "systemic"[All Fields]) OR "scleroderma"[All Fields] OR "scleroderma, localized"[MeSH Terms] OR ("scleroderma"[All Fields] AND "localized"[All Fields]) OR ("arthritis, juvenile"[MeSH Terms] OR ("arthritis"[All Fields] AND "juvenile"[All Fields])) OR ("polymyositis"[MeSH Terms] OR "polymyositis"[All Fields]) OR ("dermatomyositis"[MeSH Terms] OR "dermatomyositis"[All Fields]) OR ("spondylarthritis"[MeSH Terms] OR "spondyloarthritis"[All Fields]) OR ("fibromyalgia"[MeSH Terms] OR "fibromyalgia"[All Fields]))

The full final search strategy for each database will be documented in the final publication of the results, in alignment with the 2021 PRISMA-S extension to the PRISMA Statement for Reporting Literature Searches in Systematic Reviews [54].

### Study records

#### Data management

The search strategy (platform(s), search terms, date of each search and number of results) for each database will be documented in Word, to be reported in the final publication of the results, as recommended by PRISMA [54]. Once the searches have been performed in each database, references will be amalgamated in EndNote, and duplicates removed, before exporting into Excel. The screening of all articles will be conducted in Excel. All screening decisions at each stage will be documented, including number of articles included/excluded + reasons for exclusion, to be reported as a summary flowchart in the final publication of the results [54]. All documents will be stored within a team SharePoint, backed up on secure servers at the University of Bath.

#### Selection process

One author (RB) will be responsible for conducting the database searches, exporting into EndNote/Excel and managing all relevant study documentation. The same author will progressively screen all titles, abstracts and full texts, independently against the selection criteria, with a second author independently screening 15% of articles to check for agreement regarding eligibility. If there is no full text available for an article, a copy will be requested from the interlibrary loans service. If the full text cannot be obtained from the interlibrary loans service, the authors will be contacted for a copy of the article. Where only an abstract (but no full text) can be located, the article will ultimately be excluded, as detailed above.

All reasons for exclusion at each stage will be documented in the screening Excel. Any discrepancies between reviewers will be discussed at length and resolved by consensus or (if required) by a third independent reviewer. The independent second author screening will be repeated for an additional 5% of articles until reasonable agreement (> 95% at full-text stage) is reached.

It is anticipated that multiple publications may exist for a single identified app. For example, a research group developing and evaluating a smartphone app to support self-management of RA may have published 3 articles: (1) a qualitative study of user needs to inform app design, comprised of focus groups with people living with RA, and interviews with treating healthcare professionals; (2) a study reporting the theoretically informed, evidence-based design of a novel app to promote behaviour change, including a mapping of user needs to intervention content, BCTs and design features; and (3) a pre-post intervention study to document app acceptability, usability and effectiveness at promoting behaviour change. As such, articles pertaining to a single intervention will be grouped together before commencing data extraction. Whether or not to include all relevant articles pertaining to a single intervention, or to exclude some as duplicates, will be decided on a study-by-study basis.

#### Data collection process

For included articles, all data will be extracted utilising a data extraction table in Excel. The table will be iteratively piloted by the study team before finalisation, whereby existing data items may be amended/additional data items added, if deemed appropriate during the piloting process. For example, if other useful data items become apparent. Data will be extracted by one author (RB) and checked by a second reviewer.

Where information on outcomes of interest is not reported in an eligible publication, this will be documented within the data extraction table; its absence is in itself a finding (e.g. no clarification of theoretical framework used for development; no transparency regarding funding). As such, no attempt will be made to obtain or confirm data from the study investigators.

### Data items

The full list of data items is reported below in the draft data extraction table (Table 2).

### Outcomes and prioritisation

In alignment with the primary versus exploratory research questions above, the study outcomes are as follows:

- Primary (prioritised) outcomes include the documented theoretical frameworks of developed RMD self-management apps, intervention components (targeted behaviours, BCTs, app features), development strategies, facilitators/barriers to engagement and methods to evaluate effectiveness.
- Exploratory outcomes include an overview of the effectiveness, acceptability and usability of RMD self-management apps reported in the literature.

The selection of outcomes (as listed in Table 2) was determined based on identified gaps in the literature, EULAR guidance for developing self-management interventions for RMDs (Nikiphorou et al., 2021) and EULAR guidance for developing smartphone apps to support self-management of RMDs (Najm et al., 2019) [26, 31]. A recently published taxonomy of app features, developed according to human motivational theory (specifically, self-determination theory — Villalobos-Zúñiga et al., 2020), was also used to categorise app features [55].

**Table 2:** Data extraction table

**Summary of publication**		
**Name of intervention**		
**Study title**		
**Author(s)**		
**Year**		
**DOI or URL**		
**Protocol available online?** (Provide link)		
**Prior feasibility and pilot testing? Y/N**		
**Study design** (e.g. RCT, observational, case–control study, feasibility, pilot)		
**Methods** (quantitative, qualitative, mixed methods)		
**Aims/purpose of study**		
**Location/setting** (e.g. country, number of participating centres, primary/secondary care, specialty)		
**Demographics of population**		
**Mean age (SD)**		
**Female**: # (%)		
**Population**: Condition(s)		
**Inclusion/exclusion criteria** (copy and paste)		
**Mean duration of symptoms/time since diagnosis**		
**Integration of appropriate theory**		
**Has a published/validated development framework been cited? Y/N** (if yes, please specify, e.g. intervention mapping, person-based approach, Centre for eHealth Research and Disease Management (CeHRes) development approach)		
**Targeted health behaviour(s)**		
**Consideration of motivational/behaviour change theory to promote self-management behaviour? Y/N** (if yes, please specify theory used, e.g. self-efficacy theory, self-determination theory, MoVo process model, COM-B model of behaviour)		
**Specification of BCTs utilised and proposed mechanism(s) of action? Y/N** (if yes, please list BCTs + corresponding conceptual MoAs; requires conscious acknowledgement of techniques used and conceptual frameworks by which they promote behaviour change, e.g. an app may display autonomy-supportive language, but not have considered how or why this promotes behaviour change, or the motivational framework supporting its use)		
**Consideration of theoretical frameworks to promote app engagement? Y/N** (if yes, please specify theory or framework used, e.g. persuasive systems design, self-determination theory-based taxonomy of app features)		
**Intervention development**		
^a^**Transparency regarding funder**		
^a^**Transparency on data ownership**		
^a^**Adherence to data protection and regulatory frameworks**		
**Acknowledgement of alignment with clinical guidelines**		
**Stage of development** (e.g. pilot/feasibility testing, deployed)		
**Strategies of development** (copy overview of methodology if available)		
^a^**Co-design with patients? Y/N**		
^a^**Co-design with HCPs? Y/N** (if yes, please specify professions)		
**Co-design with others? (Y/N)** (if yes, please specify, e.g. stakeholder committee, behavioural scientists, HCI researchers)		
**Is any training/education provided to the patients** on using the technology?		
**Is any training/education provided to the HCP** on using the technology?		
**Self-management intervention content** (please specify details, list developed using guidance from EULAR (Nikiphorou et al., 2021) [26] and patient organisations [NASS, NRAS, versus arthritis])		
^a^Evidence-based, up-to-date, scientifically justifiable content		
Disease education		
Flare management		
Coping with pain		
Coping with fatigue		
Managing sleep		
Medication education		
Medication management (e.g. reminders)		
Joint decision-making		
Psychological support (e.g. cognitive behavioural therapy)		
Mental health assessment		
Physical activity/ stretching		
Physiotherapy techniques guided or created by a physiotherapist		
Occupational health (work-related) guidance		
Smoking		
Alcohol intake		
Nutrition		
Other lifestyle advice and support (please specify)		
Social support		
Clinical action plans		
Problem-solving		
Signposting to patient organisations		
Signposting to advice line/HCP contacts		
Signposting to other resources (please specify)		
^a^Tailoring/personalisation		
Other		
**APP features, according to SDT motivational taxonomy** (Villalobos-Zúñiga et al., 2020) — Y/N		
Reminders		
Goal setting		
Motivational messages		
Pre-commitment		
Activity feedback		
History (e.g. progress graph)		
Log/self-monitoring		
Rewards		
Performance sharing		
Peer challenging		
Messaging (peer to peer)		
Messaging (to HCPs — please specify asynchronous/synchronous + purpose)		
^a^Communication moderation		
Others (please specify)		
Operating system (i.e. iOS or Android)		
**Barriers/facilitators to engagement**		
Barriers to engagement with digital intervention		
Barriers to engagement with self-management behaviours		
Facilitators of engagement with digital intervention		
Facilitators of self-management behaviours		
**Measures of effectiveness**		
Self-management support measures, e.g. patient activation, self-efficacy		
What objective outcomes are used (e.g. accelerometer data, spinal mobility?		
What clinician-reported outcome measures (ClinROs), diaries, or other tools are used?		
What patient-reported outcome measures (PROMs) are used?		
What is used to measure intervention adherence?		
What is used to measure intervention acceptability?		
What is used to measure intervention usability?		
At what time points are outcomes assessed?		
**Results/description of effectiveness**		
Was the intervention effective (*qualitative*)?		
Was the intervention effective (*quantitative*)?		
Adherence?		
Acceptability?		
^a^Usability? (Across all ages, abilities)		
^a^Cost-effectiveness?		
Contextual influences on intervention success (economic factors, available resources, local healthcare system structure) [53]		
Conclusions, clinical implications, future directions?		
Limitations (of the intervention)		
**Methodological strengths/limitations for evaluation of effectiveness**		
Study design		
Strengths (of the study design)		
Limitations (of the study design)		

*NASS* National Axial Spondyloarthritis Society, *NRAS* National Rheumatoid Arthritis Society

^**a**^From EULAR points to consider for the development, evaluation and implementation of mobile health applications aiding self-management in people living with RMDs [31]

### Risk of bias in individual studies

All publications selected for inclusion in the systematic literature review will undergo rigorous critical appraisal by one independent reviewer (with prior quality assessment experience [56]) using the appropriate tool from The Joanna Briggs Institute (JBI) [57, 58, 59]. The suite of JBI tools were deemed most appropriate for this systematic literature review, due to the broad range of tools available to assess various study designs/ evidence types (both quantitative and qualitative – ensuring consistency in our assessment), and where appropriate, these tools have been recently updated in order to align with current methodological developments/ nomenclature in this field, including the concept of “risk of bias” (i.e., potential for systematic error) rather than “critical appraisal” to interrogate quantitative evidence. These assessment tools are recommended and deemed acceptable [60]. Information on study strengths/limitations (as reported in the literature) will also be captured in the data extraction table.

#### Meta-biases

For the purposes of our research question, a formal assessment of publication bias (e.g. assessed by funnel plots and Egger’s test), as would be done for a meta-analysis, is not appropriate. However, a narrative consideration of publication bias will be provided in the final results report. For those studies evaluating intervention effectiveness, acceptability or usability, outcome reporting bias will be assessed through comparison of reported outcomes in the results, versus those listed in the methods and/or study protocol, if available [51]. Language bias, a subtype of reporting bias, will also be discussed in the results, including a summary of the number of studies excluded on this basis (i.e. study not published in English), and the potential implications/ consequences of exclusion [61].

### Data synthesis

#### Systematic, narrative synthesis

A systematic, narrative synthesis of evidence will be presented, based on data items captured in the data extraction table [53]. This analysis will involve descriptive data, for example frequency counts of the target population of documented apps, or of specific BCTs and theoretical frameworks utilised (e.g. *n*(%) apps targeted a specific condition; the most commonly used BCT was X, reported in the literature for *n*(%) of identified apps; *n*(%) of publications considered motivational theory during app development, the most common cited theory was X, reported in n publications). Results will be organized based on the outcomes and headings presented in Table 2. The findings will be reported in the text, and summarised in tables, where appropriate, as done similarly by Najm et al. (2019) [47]. Relationships and findings will be explored both within and between the included studies, in alignment with the guidance from Popay et al. (2006) [62]. The updated 2020 PRISMA guidelines will be followed when reporting the results from this review to ensure complete and transparent reporting [63].

#### Meta-summary of qualitative findings regarding barriers/facilitators to intervention engagement

If appropriate (depending on quality and pool of evidence identified [53]), qualitative meta-summary techniques, as first described by Sandelowski and Barroso, will be used to combine and summarise qualitative findings regarding barriers/facilitators to (a) engagement with self-management behaviour and (b) engaging with smartphone self-management apps, for people living with RMDs [64]. This methodology has been similarly reported by Herber et al. (2017) when exploring barriers and facilitators to self-care in heart failure patients [65]. The approach includes three phases: (1) extraction of relevant findings from each publication (via Table 2), (2) reduction of these statements into abstracted findings and (3) calculation of effect sizes (number of publications containing the finding/by the total number of publications reporting barriers/facilitators) [64]. Caution will be taken when reporting effect sizes, will require review and interpretation by relevant stakeholders (people living with RMDs and treating HCPs) and be presented in the context of the search strategy for the present study. Nevertheless, reporting of effect sizes for these qualitative findings allows for a “quantitative transformation of qualitative data” and potentially allows for a weighting of a finding in relation to acting as a barrier or a facilitator to self-management/app engagement, useful for the development of future interventions [64, 66, 67].

### Confidence in cumulative evidence

As an exploratory, narrative study (with no meta-analysis/analysis of treatment efficacy), confidence in cumulative evidence will not be formally assessed using, for example Grading of Recommendations Assessment, Development and Evaluation working group methodology. However, confidence in our results and applicability to other populations will be described pragmatically, including consideration of risk of bias across studies, inconsistency and publication bias. We will also provide a tabular summary of the types of evidence included, to provide context to our findings.

## Discussion

Self-management is an essential component of patient care for chronic conditions such as RMDs, whereby patients must be supported to manage the life-long practical, physical and psychological impacts of their disease [26, 68, 69]. However, post-pandemic workload pressures have led to many services struggling to deliver specialist support to patients, particularly regarding education and non-pharmacological treatment, making good long-term self-management more difficult for patients [20]. There is therefore an unmet need for novel, scalable interventions that can better support patients in their self-management and reduce pressure on healthcare services. Digital self-management interventions delivered via smartphone apps could provide a solution to fulfil this currently unmet need.

To optimise patient engagement with smartphone self-management apps and the health behaviours they are trying to promote, researchers and designers should leverage theoretical frameworks from psychology and computer science during app development [34–36, 70]. However, it is not yet known how motivational theories and published/validated development frameworks are being applied to inform app development in rheumatology. This review aims to fill this evidence gap, by collating and summarising the current published scientific literature on smartphone apps developed to support the self-management of RMDs. The results of this systematic literature review will provide insights for healthcare professionals, researchers, app designers and policy makers, to inform future development and implementation of smartphone apps. Evidence gaps for future research will also be identified. Findings will be disseminated through a final manuscript/publication of results and via a conference abstract, patient organisations and social media.

### Anticipated challenges

Smartphone self-management apps for RMDs can be considered complex interventions, due to the variety of intervention components and app features (promoting a range of self-management behaviours/app engagement) and the context with which they are deployed (healthcare setting, for a heterogeneous groups of diseases) [71]. As outlined above, guidance from Petticrew et al., regarding synthesis of information from complex interventions, will therefore be followed when displaying final results [53]. This review will collect and synthesise a substantial amount of information (Table 2). As such, displaying results may be challenging, and formulating systematic, yet creative, easy-to-interpret tables/graphs will be critical.

As a lot of complex information is to be collected/synthesised, we anticipate that there may not be scope to discuss all results in detail in the final publication. Therefore, to ensure transparency and omit reporting bias, the final completed data extraction Excel documenting all extracted findings from each article will be attached as an appendix to the final resulting publication. We may also consider separating the write-up of our results into two separate publications to distinguish reporting of intervention development versus how effectiveness has been evaluated, if appropriate based on the research findings, to ensure that we cover each of our study objectives in sufficient detail without compromising readability of the final publication.

There are over 200 RMDs. Although our tested search strategy has provided a reasonable number of identified articles, if ultimately too many publications are eligible for inclusion (> 100), we may be forced to reduce our population to inflammatory RMDs only. Results for degenerative RMDs could be reported in a separate publication at a later date.

### Strengths and limitations

The proposed systematic literature review will provide a timely and comprehensive overview of the development strategies, interventional components and theoretical underpinnings of existing smartphone apps, designed to support the self-management of RMDs, thus filling an important evidence gap in the rheumatology field. A key strength of this review is the choice of multiple databases from which to identify publications. A recent study identified optimal database combinations for literature searches in systematic reviews and estimated that the combination of Embase and MEDLINE and either Google Scholar or Web of Science could be regarded as sufficient, with 96% recall [72]. This in alignment with our chosen databases, whereby searches will be run in PubMed, Embase, MEDLINE, Web of Science, Scopus and PsycINFO. The search strategy for this review was first developed in PubMed and then adapted to Scopus, Web of Science, Embase, MEDLINE and PsycINFO. These databases use different thesauruses to index key search terms (e.g. MeSH in PubMed, Emtree in Embase) and have different available functionalities for searching (e.g. truncation, wildcards). The final search string/syntax will therefore differ for each database, based on the use of different keywords/index terms/functions. Nevertheless, the search strategy was developed iteratively and collaboratively within a team of experienced researchers, a consultant rheumatologist and an expert librarian. Initial search terms were developed from a prior systematic literature review conducted by EULAR to inform their 2019 recommendations for the development, evaluation and implementation of mobile health applications aiding self-management in people living with RMDs [31, 47]. The full final search strategy for each database will be documented in the results of the final publication.

There is large heterogeneity both within and between different RMD diagnoses, which may thus require different approaches to self-management and intervention design and delivery. The scope of this review was therefore limited to chronic RMDs only. Literature pertaining to smartphone apps designed for patients with non-specific musculoskeletal disorders such as low back pain, acute musculoskeletal disorders as result of injury or interventions designed to support patients pre-/post-surgery will not be captured. An exploration of the literature for these conditions may be warranted in future. The heterogeneity of the population of interest for this study will be acknowledged and discussed within the final publication of our results, with the distinction made between typically conceptualised degenerative and inflammatory chronic RMDs.

Our methodology involves eligibility screening by one reviewer, with 15% of identified publications independently screened by a second reviewer to check for agreement. The independent second author screening will be repeated for an additional 5% of articles until reasonable agreement (> 95% at full-text stage) is reached. This chosen approach aims to maximise efficiency and resourcing, without compromising reliability. Nevertheless, the risk of missing relevant studies will remain higher than if all records were checked by two or more independent reviewers [73].

Finally, although some information on effectiveness of identified interventions will be captured, this is not a primary objective for the study, and meta-analytical techniques to assess and summarise effectiveness (or potential effect modifiers/covariates) will not be utilised. This should be investigated in future studies, including exploration/consideration of how systematic use of specific theoretical frameworks, behaviour change techniques or persuasive design features may underpin or modify intervention effects.

## Supplementary Information

**Additional file 1.** PRISMA-P (Preferred Reporting Items for Systematic review and Meta-Analysis Protocols) 2015 checklist: recommended items to address in a systematic review protocol*.

## Data Availability

All data generated or analysed during this study will be included in the final published article of the results (and its supplementary information files).

## Abbreviations

ACR: American College of Rheumatology
BCT: Behaviour change technique
BSR: British Society for Rheumatology
EULAR: The European Alliance of Associations for Rheumatology
JBI: Joanna Briggs Institute
NASS: National Axial Spondyloarthritis Society
NRAS: National Rheumatoid Arthritis Society
OA: Osteoarthritis
PICO: Population, intervention, comparator and outcome
PRISMA: Preferred reporting items for systematic review and meta-analysis
PRISMA-P: Preferred reporting items for systematic review and meta-analysis protocols
PRISMA-S: PRISMA statement for reporting literature searches in systematic reviews
PROSPERO: International prospective register of systematic reviews
RA: Rheumatoid arthritis
RMD: Rheumatic and musculoskeletal disease
SpA: Spondyloarthritis
